# Prevalence trends in respiratory symptoms and asthma in relation to smoking - two cross-sectional studies ten years apart among adults in northern Sweden

**DOI:** 10.1186/1939-4551-7-1

**Published:** 2014-01-02

**Authors:** Helena Backman, Linnea Hedman, Sven-Arne Jansson, Anne Lindberg, Bo Lundbäck, Eva Rönmark

**Affiliations:** 1 The OLIN Studies, Department of Research and Development, Norrbotten County Council, Luleå, Sweden; 2 Division for Occupational and Environmental Medicine, Department of Public Health and Clinical Medicine, Umeå University, Umeå, Sweden; 3 Division for Respiratory Medicine and Allergy, Department of Public Health and Clinical Medicine, Umeå University, Umeå, Sweden; 4 Krefting Research Centre/Department of Internal Medicine, The Sahlgrenska Academy, University of Gothenburg, Gothenburg, Sweden

**Keywords:** Respiratory symptoms, Asthma, Prevalence, Smoking, Attributable risk, Trends

## Abstract

**Background:**

Smoking is considered to be the single most important preventable risk factor for respiratory symptoms. Estimating prevalence of respiratory symptoms is important since they most often precede a diagnosis of an obstructive airway disease, which places a major burden on the society. The aim of this study was to estimate prevalence trends of respiratory symptoms and asthma among Swedish adults, in relation to smoking habits. A further aim was to estimate the proportion of respiratory symptom and asthma prevalence attributable to smoking.

**Methods:**

Data from two large-scale cross-sectional surveys among adults performed in northern Sweden in 1996 and 2006 were analysed. Identical methods and the same questionnaire were used in both surveys. The association between smoking, respiratory symptoms and asthma was analysed with multiple logistic regression analyses. Changes in prevalence of respiratory symptoms and asthma from 1996 to 2006 were expressed as odds ratios. Additionally, the population attributable risks of smoking were estimated.

**Results:**

The prevalence of most respiratory symptoms decreased significantly from 1996 to 2006. Longstanding cough decreased from 12.4 to 10.1%, sputum production from 19.0 to 15.0%, chronic productive cough from 7.3 to 6.2%, and recurrent wheeze from 13.4 to 12.0%. Any wheeze and asthmatic wheeze remained unchanged. This parallels to a decrease in smoking from 27.4 to 19.1%. In contrast, physician-diagnosed asthma increased from 9.4 to 11.6%. The patterns were similar after correction for confounders. All respiratory symptoms were highly associated with smoking, and the proportion of respiratory symptoms in the population attributed to smoking (PAR) ranged from 9.8 to 25.5%. In 2006, PAR of smoking was highest for recurrent wheeze (20.6%).

**Conclusions:**

In conclusion, we found that respiratory symptoms, in particular symptoms common in bronchitis, decreased among adults in northern Sweden, parallel to a decrease in smoking from 1996 to 2006. In contrast, the prevalence of physician-diagnosed asthma increased during the same time-period. Up to one fourth of the respiratory symptom prevalence in the population was attributable to smoking.

## Background

Tobacco consumption is one of the main risk factors of premature death and disability worldwide [1]. The use of tobacco is rising globally mainly because of an increasing smoking prevalence in many low and middle income countries [2]. The societal costs of diseases related to tobacco use are substantial; e.g. estimated to more than €500 billion in 2009, which corresponds to 4.6% of the Gross Domestic Product of the European Union [3]. In 2008, about one out of three adults in the world smoked [4], and the overall pattern in the World Health Organisation (WHO) European region was similar. In Sweden however, the prevalence of smoking has decreased during the last decades [5,6].

Smoking is considered to be the single most important preventable risk factor for respiratory symptoms, chronic bronchitis and chronic obstructive pulmonary disease (COPD) [7]. The relationship between asthma and smoking is unclear, and contradictory results have been found in different studies. Smoking has been considered a risk factor for incident asthma in some studies [8-10], while others have found no association [11]. Some cross-sectional studies on asthma prevalence have found an association between ex-smoking or ever-smoking and asthma [6,12], while others have found no such association [13,14].

Asthma, chronic bronchitis and COPD contribute a major burden on the society in terms of disability, mortality and costs [15-17]. The prevalence of asthma has been increasing from the 1950s [18-21], however, recent studies imply that the asthma increase might have reached a plateau in several westernized countries [6,22,23]. Worldwide, the burden of COPD is steadily increasing [24]. Estimating prevalence of respiratory symptoms is important since symptoms most often precede a diagnosis of an obstructive airway disease [7], and since symptom prevalence less likely is biased by healthcare practice or diagnostic activity compared to diagnoses of obstructive airway diseases. Few recent studies among adults have reported trends in prevalence of respiratory symptoms and asthma.

Within the Obstructive Lung Disease in Northern Sweden (OLIN) Studies, prevalence and incidence of obstructive airway diseases have been studied since 1985 [25]. The aim of the present study was to estimate prevalence trends of respiratory symptoms and asthma among adults in relation to smoking habits by two large-scale cross-sectional studies performed in 1996 and 2006. A further aim was to estimate the proportion of respiratory symptom and asthma prevalence attributable to smoking.

## Methods

### Study population

This study was performed in Norrbotten, the northernmost county of Sweden. In 1996, a population-based sample of 8,333 subjects in ages 20–69 years were sent a postal questionnaire including questions about respiratory symptoms, diseases and smoking habits. The sample was randomly selected from the Swedish population register. After two reminders, 85% participated.

Correspondingly, a new random sample consisting of 7,997 subjects in ages 20–69 years were invited in 2006. The methodology and questionnaire were identical with the 1996 survey and 77% participated (Table 1). The total population in Norrbotten between 20–69 years of age was approximately 168,000 in 1996 and 159,000 in 2006.

**Table 1:** Study population, invited and participants by study year, age group and sex

		**Age group**					**Sex**		
		**20-29y**	**30-39y**	**40-49y**	**50-59y**	**60-69y**	**Women**	**Men**	**All**
Study year 1996									
Invited	N	1619	1721	1895	1694	1404	3982	4351	8333
Participated	n	1317	1429	1616	1490	1252	3471	3633	7104
	%	81.3	83.0	85.3	88.0	89.2	87.2	83.5	85.3
Study year 2006									
Invited	N	1428	1514	1718	1820	1517	3843	4154	7997
Participated	n	941	1080	1312	1510	1322	3135	3030	6165
	%	65.9	71.3	76.4	83.0	87.1	81.6	72.9	77.1

### Questionnaire

The questionnaire includes questions on respiratory symptoms and diseases, demographic data, smoking and other possible determinants of disease. It was developed from the British Medical Research Council questionnaire and has been used in several large-scale studies mainly in Sweden, Finland and Estonia [6,9,22,25-28].

### Definitions

#### Asthma variables

*Physician-diagnosed asthma:* Have you been diagnosed as having asthma by a physician. *Ever asthma:* Do you have, or have you ever had asthma. *Asthma medication:* Do you use asthma medication (on a regular basis or when needed).

#### Bronchitis variables

*Longstanding cough:* Have you had longstanding cough during recent years. *Sputum production:* Do you usually have phlegm when coughing, or do you have phlegm in your chest that is difficult to bring up. *Chronic productive cough:* Bringing up phlegm when coughing on most days during periods of at least 3 months during at least 2 successive years.

#### Wheeze variables

*Any wheeze:* Have you at any time during the last 12 months had wheezing or whistling in your chest. *Recurrent wheeze:* Do you usually have wheezing or whistling in your chest when breathing. *Asthmatic wheeze:* Wheeze with breathlessness during the last 12 months when you did not have a cold.

#### Other variables

*Family history of asthma:* Does any of your parents or siblings have, or have had, asthma. *Smokers* reported smoking during the 12 months preceding the survey, while *Ex-smokers* reported having quit smoking at least 12 months prior to the study. Smokers answered a question about the number of consumed cigarettes: <5 cigarettes per day, 5–14 cigarettes per day, or >14 cigarettes per day. *Non-smokers* reported neither smoking nor ex-smoking.

### Ethical approval

The study was approved by the Regional Ethical Review Board at Umeå University, Sweden.

### Analysis

Statistical analyses were performed using the IBM SPSS software (Version 20.0; SPSS Inc., Chicago IL, USA). Differences in prevalence between groups were tested by the chi-square test or Mantel-Haenszel test for trend and differences in continuous variables were tested by a two-tailed Student’s T-test. A p-value <0.05 was considered statistically significant. Multiple logistic regression models were used in order to calculate Odds Ratios (OR:s) with 95% confidence intervals (CI) for the simultaneous effects of independent variables on different outcomes. The independent variables in the logistic regression models were; age, sex, smoking habits, family history of asthma and year of study, where age was divided into 10-year categories. The same model was also applied for each study year separately (each survey).

The population attributable risk (PAR) in% of a certain risk factor expresses the proportion of the prevalence of a condition in the population which is attributable to that risk factor. PAR of smoking was calculated each year as: ((P_T_-P_0_)/P_T_)*100, where P_T_ = Prevalence of a symptom/disease among all and P_0_ = Prevalence of a symptom/disease among non- and ex-smokers. Corresponding 95% CI for PAR were calculated using the Maximum Likelihood method [29].

PAR was also calculated based on Odds Ratios for smoking (non- and ex-smoking as reference) from logistic regression models adjusted for age, sex and family history of asthma, and is then referred to as “adjusted PAR”. The formula for adjusted PAR is as follows: ((OR-1)/OR)*P*100, where P = Prevalence of smoking among cases with the symptom/disease [30].

## Results

### Participation

The participation rate was significantly higher in 1996 compared to 2006 in most age groups and among men and women. In 1996, 7,104 adults (85%) participated and in 2006, 6,165 (77%) participated. In both surveys, the participation rates were higher among women compared to men, and increased by age (Table 1).

### Smoking habits

The prevalence of smoking decreased significantly from 27.4% in 1996 to 19.1% in 2006 (p < 0.001). The decrease was significant in all age groups as well as among men and women (Table 2). Additionally, the proportion of smokers who smoked >14 cigarettes/day decreased among both smoking women (from 21.5 to 17.9%, p = 0.073) and men (from 35.2 to 26.0%, p = 0.001).

**Table 2:** Prevalence (%) of smoking habits by study year, age group and sex

		**Age group**					**Sex**			
	**n All**	**20-29y**	**30-39y**	**40-49y**	**50-59y**	**60-69y**	**Women**	**Men**	**All**	**All**^**¤**^
Smoking habits 1996										
Non-smokers	3600	69.3	55.0	41.3	42.0	50.8	51.9	50.3	51.1	51.5
Ex-smokers	1511	9.3	17.9	24.9	26.5	27.8	18.6	24.1	21.5	21.1
Smokers	1932	21.4	27.1	33.7	31.5	21.3	29.4	25.5	27.4	27.4
<5 cig/day	402	7.3	7.0	5.9	5.0	3.3	5.1	6.3	5.7	5.8
5-14 cig/day	944	10.5	13.0	17.0	15.1	10.4	17.3	9.7	13.4	13.4
>14 cig/day	523	2.9	6.3	10.2	10.4	6.4	6.1	8.7	7.4	7.3
Missing data	63	0.8	0.8	0.7	1.0	1.2	0.9	0.9	0.9	0.9
Smoking habits 2006										
Non-smokers	3538	75.8	74.0	56.8	45.6	48.1	55.2	61.2	58.1	59.1
Ex-smokers	1372	7.8	13.3	20.5	30.1	34.0	22.0	23.1	22.5	21.8
Smokers	1178	16.4	12.7	22.7	24.3	17.9	22.8	15.7	19.3	19.1
<5 cig/day	279	7.2	3.6	4.7	4.6	3.4	4.8	4.4	4.6	4.6
5-14 cig/day	609	6.8	6.1	11.5	13.8	9.6	13.2	6.6	10.0	9.8
>14 cig/day	237	1.7	2.5	5.5	4.9	3.8	3.9	3.9	3.9	3.8
Missing data	53	0.6	0.6	0.9	0.9	1.2	0.8	0.9	0.9	0.9
P-value*		0.003	<0.001	<0.001	<0.001	0.002	<0.001	<0.001	<0.001	

All^¤^ = Standardized to the age and gender distribution in Norrbotten each year.

*Chi-square test of difference in prevalence of smoking habits in terms of non-smokers, ex-smokers and smokers between surveys.

Ca 1% lacks data of smoking habits each year.

Smoking habits differed significantly (p < 0.001) between age groups in both surveys, with smoking being most common among subjects 40–59 year old (Table 2). In both surveys, more women than men were smokers (p < 0.001).

### Respiratory symptoms and asthma

**Figure 1:**
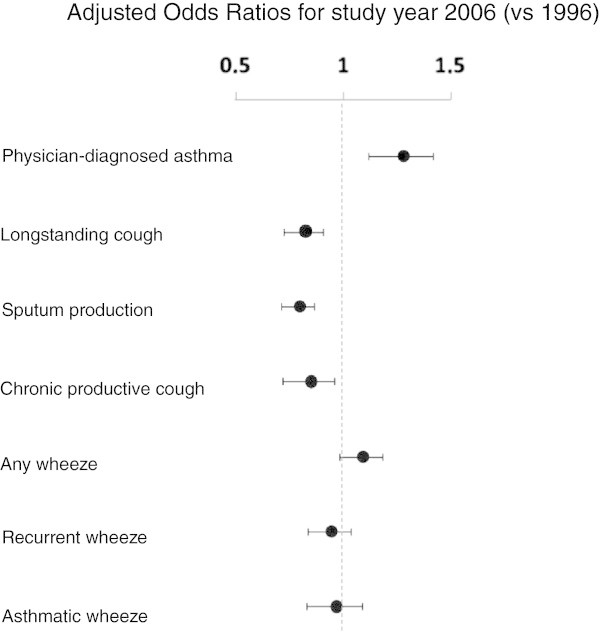
**Changes in asthma and respiratory symptom prevalence from 1996 to 2006.** Legend: The changes in physician-diagnosed asthma and respiratory symptom prevalence from 1996 to 2006 are expressed as Odds Ratios with corresponding 95% Confidence Intervals for 2006 as survey year (1996 as reference) from logistic regression models adjusted for age group, sex, family history of asthma and smoking habits.

The prevalence of most respiratory symptoms decreased significantly from 1996 to 2006. Longstanding cough decreased from 12.4 to 10.1%, sputum production from 19.0 to 15.0%, chronic productive cough from 7.3 to 6.2% and recurrent wheeze from 13.4 to 12.0%. Any wheeze and asthmatic wheeze remained unchanged. In contrast, the prevalence of ever asthma increased significantly from 10.1 to 13.4%, physician-diagnosed asthma from 9.4 to 11.6% and the use of asthma medication from 10.9 to 12.4% (Table 3). The change in prevalence from 1996 to 2006 expressed as odds ratios is displayed in Figure 1. A significant decrease was found for longstanding cough (OR 0.82, 95% 0.74-0.92), sputum production (OR 0.80, 95% 0.72-0.87) and chronic productive cough (OR 0.85, 95% 0.74-0.98), while physician-diagnosed asthma increased (OR 1.28, 95% 1.14-1.44).

**Table 3:** Prevalence (%) of respiratory symptoms and asthma by study year, age group and sex

		**Age group**						**Sex**					
	**Year**	**20-29y**	**30-39y**	**40-49y**	**50-59y**	**60-69y**	P-value*	**Women**	**Men**	P-value**	**All**	**All**^**¤**^	P-value***
**Asthma variables**													
Ever asthma	1996	12.8	11.5	8.9	7.2	10.5	<0.001	10.4	9.7	0.318	10.1	10.1	
	2006	18.3	14.0	12.6	12.5	10.9	<0.001	14.3	12.2	0.016	13.3	13.4	<0.001
Physician-diagnosed	1996	11.8	10.1	7.7	6.8	10.7	0.016	9.4	9.3	0.899	9.3	9.4	
asthma	2006	15.6	12.1	10.8	10.6	9.5	<0.001	12.4	10.5	0.020	11.5	11.6	<0.001
Asthma medication	1996	11.8	11.8	9.5	10.0	11.7	0.395	12.2	9.7	0.001	10.9	10.9	
	2006	14.1	11.9	11.7	12.6	12.0	0.315	13.7	11.0	0.001	12.4	12.4	0.009
**Bronchitis variables**													
Longstanding cough	1996	13.0	12.0	12.0	11.9	13.3	0.883	13.6	11.2	0.002	12.4	12.4	
	2006	10.6	9.0	9.1	11.1	10.7	0.334	11.0	9.2	0.017	10.1	10.1	<0.001
Sputum production	1996	19.9	19.4	15.8	19.1	22.0	0.296	19.0	19.2	0.830	19.1	19.0	
	2006	18.6	14.1	12.2	15.4	15.4	0.288	14.7	15.2	0.551	15.0	15.0	<0.001
Chronic productive	1996	4.9	5.9	5.7	9.3	11.9	<0.001	7.1	7.8	0.260	7.4	7.3	
cough	2006	6.1	5.1	5.5	6.8	7.7	0.017	5.3	7.3	0.001	6.3	6.2	0.009
**Wheeze variables**													
Any wheeze	1996	18.4	19.1	16.5	19.0	20.3	0.295	19.6	17.6	0.030	18.6	18.5	
	2006	20.8	17.7	17.5	19.7	17.6	0.317	19.0	18.2	0.423	18.6	18.6	0.974
Recurrent wheeze	1996	12.1	13.6	12.2	13.8	15.6	0.021	14.1	12.8	0.119	13.4	13.4	
	2006	10.3	11.1	11.8	13.8	12.3	0.030	11.9	12.2	0.765	12.1	12.0	0.019
Asthmatic wheeze	1996	7.8	7.8	6.3	8.1	9.6	0.117	7.6	8.1	0.446	7.9	7.8	
	2006	8.3	5.8	7.3	8.2	7.1	0.857	7.6	7.2	0.519	7.4	7.4	0.305

All^¤^ = Standardized to the age and gender distribution in Norrbotten each year.

*Test for trend by age group.

**Chi-square test of difference in prevalence by sex.

***Chi-square test of difference in prevalence by year of study (among All).

Also among subjects with physician-diagnosed asthma, the proportion reporting symptoms common in asthma decreased significantly from 1996 to 2006; any wheeze from 72.1 to 63.7% (p = 0.001), recurrent wheeze from 62.2 to 51.0% (p < 0.001), asthmatic wheeze from 42.9 to 32.9% (p < 0.001) and use of asthma medication from 80.2 to 75.5% (p = 0.036).

**Figure 2:**
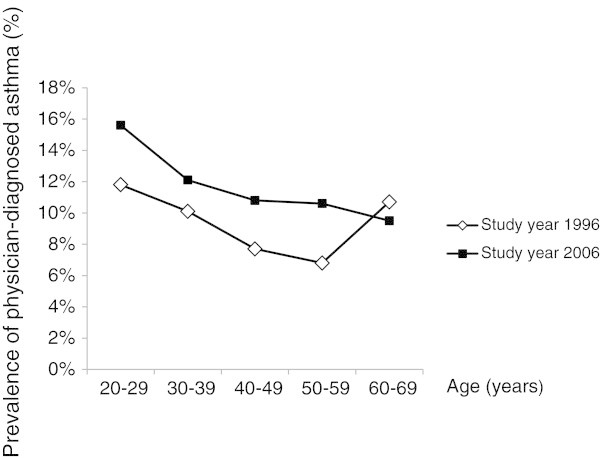
Prevalence (%) of physician-diagnosed asthma by age group and study year.

The prevalence of physician-diagnosed asthma decreased with increasing age in both surveys (except in the age group 60–69 years in 1996) (Figure 2), while the prevalence of chronic productive cough and recurrent wheeze increased with increasing age. The prevalence of most symptoms and use of asthma medication was higher among women than men in both surveys (Table 3).

### Respiratory symptoms and asthma in relation to smoking

Among non-smokers, ex-smokers, as well as among smokers, the prevalence of most asthma variables increased significantly from 1996 to 2006. The prevalence of bronchitis variables did not differ between 1996 and 2006 among smokers or ex-smokers, but decreased significantly among non-smokers. The prevalence of wheeze variables did not differ significantly between the surveys when stratified by smoking habits, except for an increase in any wheeze among smokers (Table 4).

**Table 4:** Prevalence (%) of respiratory symptoms and asthma by study year and smoking habits

		**Non-smokers**		**Ex-smokers**		**Smokers**		
	**Year**	**%**	**Diff by year**	**%**	**Diff by year**	**%**	**Diff by year**	**Diff by smoking**
			**P-value**		**P-value**		**P-value**	**P-value**
**Asthma variables**								
Ever asthma	1996	9.9		11.1		9.4		0.257
	2006	12.3	0.002	15.0	0.002	14.3	<0.001	0.021
Physician-diagnosed	1996	9.0		11.0		8.3		0.023
asthma	2006	10.6	0.023	12.0	0.053	11.8	0.001	0.026
Asthma medication	1996	10.7		12.0		10.5		0.322
	2006	11.2	0.453	14.8	0.026	12.7	0.058	0.003
**Bronchitis variables**								
Longstanding cough	1996	11.7		10.0		15.6		<0.001
	2006	7.8	<0.001	10.8	0.485	16.1	0.712	<0.001
Sputum production	1996	16.1		16.9		26.3		<0.001
	2006	12.1	<0.001	14.9	0.157	23.7	0.098	<0.001
Chronic productive cough	1996	6.4		6.4		10.2		<0.001
	2006	5.1	0.027	6.6	0.879	9.3	0.436	<0.001
**Wheeze variables**								
Any wheeze	1996	14.4		17.0		27.6		<0.001
	2006	14.2	0.836	19.0	0.175	31.2	0.029	<0.001
Recurrent wheeze	1996	9.5		12.4		21.4		<0.001
	2006	8.6	0.156	13.0	0.668	21.1	0.848	<0.001
Asthmatic wheeze	1996	6.1		8.5		10.8		<0.001
	2006	5.7	0.469	9.0	0.591	10.5	0.834	<0.001

From 1996 to 2006, the proportion of smokers decreased also among symptomatics; i.e. from 37.7 to 28.8% (p = 0.005) among subjects with chronic productive cough, from 37.9 to 30.6% (p < 0.001) among subjects with sputum production, from 34.6 to 31.0% (p = 0.151) among subjects with longstanding cough, from 43.8 to 34.1% (p < 0.001) among subjects with recurrent wheeze, from 37.5 to 27.7% (p = 0.001) among subjects with asthmatic wheeze and from 24.7 to 19.9% (p = 0.035) among subjects with physician-diagnosed asthma.

All respiratory symptoms were significantly and strongly related to smoking in both surveys. For the asthma variables, the highest prevalence was found among ex-smokers (Table 4). Smoking and ex-smoking were consistently associated with recurrent wheeze in both surveys (Table 5). The OR:s increased with increased cigarette consumption, and the highest OR:s (>3) were found for recurrent wheeze and chronic productive cough among those who smoked >14 cigarettes/day. Physician-diagnosed asthma was significantly associated with ex-smoking with an OR of 1.33 in 1996 and 1.41 in 2006.

**Table 5:** Risk factor analysis: Odds Ratios with 95% Confidence Intervals from logistic regression models

		**Dependent variables**											
		**Chronic productive cough**				**Physician-diagnosed asthma**				**Recurrent wheeze**			
		**Study year 1996**		**Study year 2006**		**Study year 1996**		**Study year 2006**		**Study year 1996**		**Study year 2006**	
**Independent variables**		**OR**	**95% CI**	**OR**	**95% CI**	**OR**	**95% CI**	**OR**	**95% CI**	**OR**	**95% CI**	**OR**	**95% CI**
Age	20-29 years	1		1		1		1		1		1	
	30-39 years	1.14	(0.82-1.60)	0.87	(0.59-1.28)	0.82	(0.65-1.05)	0.78	(0.60-1.01)	1.04	(0.83-1.31)	1.18	(0.88-1.58)
	40-49 years	1.01	(0.73-1.42)	0.85	(0.59-1.22)	**0.60**	**(0.46-0.77)**	**0.64**	**(0.50-0.83)**	0.82	(0.65-1.04)	1.07	(0.81-1.41)
	50-59 years	**1.80**	**(1.32-2.47)**	1.07	(0.76-1.52)	**0.55**	**(0.42-0.72)**	**0.62**	**(0.48-0.80)**	1.03	(0.81-1.29)	1.25	(0.96-1.64)
	60-69 years	**2.59**	**(1.90-3.53)**	1.35	(0.95-1.92)	0.89	(0.69-1.15)	**0.60**	**(0.46-0.79)**	**1.31**	**(1.04-1.65)**	1.23	(0.93-1.63)
Sex	Female	1		1		1		1		1		1	
	Male	1.17	(0.98-1.41)	**1.55**	**(1.25-1.92)**	1.05	(0.89-1.24)	0.90	(0.77-1.06)	0.98	(0.85-1.13)	**1.19**	**(1.01-1.39)**
Family history of asthma	No/Don’t know	1		1		1		1		1		1	
	Yes	**1.81**	**(1.49-2.21)**	**2.37**	**(1.91-2.95)**	**3.26**	**(2.76-3.85)**	**3.39**	**(2.88-4.00)**	**2.41**	**(2.07-2.79)**	**2.71**	**(2.30-3.20)**
Smoking habits	Non-smokers	1		1		1		1		1		1	
	Ex-smokers	0.89	(0.69-1.14)	1.17	(0.89-1.54)	**1.33**	**(1.09-1.64)**	**1.41**	**(1.15-1.72)**	**1.34**	**(1.10-1.62)**	**1.50**	**(1.22-1.85)**
	<5 cig/day	0.60	(0.35-1.02)	1.41	(0.87-2.28)	1.01	(0.70-1.46)	1.09	(0.74-1.61)	**1.70**	**(1.26-2.30)**	**2.18**	**(1.55-3.06)**
	5-14 cig/day	**1.43**	**(1.09-1.86)**	**1.61**	**(1.14-2.27)**	0.85	(0.65-1.12)	**1.33**	**(1.01-1.74)**	**2.61**	**(2.14-3.17)**	**3.09**	**(2.45-3.91)**
	>14 cig/day	**3.07**	**(2.35-4.02)**	**3.31**	**(2.23-4.91)**	0.95	(0.67-1.34)	0.68	(0.41-1.14)	**3.49**	**(2.77-4.40)**	**3.32**	**(2.39-4.63)**

Statistically significant Odds Ratios (OR) with corresponding 95% Confidence Intervals (CI) are bolded.

### Population attributable risk (PAR) of smoking

**Figure 3:**
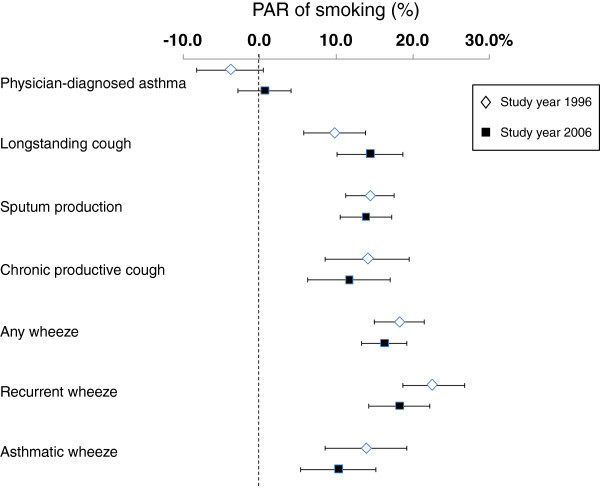
Population attributable risks (PAR in % with corresponding 95% Confidence Intervals) of smoking for physician-diagnosed asthma and respiratory symptoms in 1996 and 2006, respectively.

The PAR of smoking decreased slightly from 1996 to 2006 for all symptoms but longstanding cough. PAR was highest for recurrent wheeze both years (22.6% in 1996, 18.3% in 2006), and ranged from 9.8% to 14.1% for the bronchitis symptoms. PAR of smoking was not significant for physician-diagnosed asthma (Figure 3). In both surveys, adjusted PAR tended to be higher than PAR. Adjusted PAR was highest for recurrent wheeze (25.5% in 1996, 20.6% in 2006), and ranged from 10.9% to 18.0% for the bronchitis symptoms.

## Discussion

The main result of our study is the decrease in prevalence of most respiratory symptoms, particularly symptoms of bronchitis, from 1996 to 2006. This decrease parallels a 30% decrease in smoking prevalence, a decrease which was significant in all age groups, in both sexes, and in the proportion of smokers consuming >14 cigarettes/day. All respiratory symptoms were associated with smoking, and the proportion of respiratory symptoms attributed to smoking (PAR of smoking) varied from 10 to 25%. The decrease in smoking prevalence was greater than the decrease in prevalence of symptoms, and a tendency of a decrease in PAR of smoking from 1996 to 2006 was observed for most respiratory symptoms. In contrast to respiratory symptoms, the prevalence of physician-diagnosed asthma increased and asthma was not consistently associated with smoking.

It is of great importance for preventive decisions to identify effects of changes in smoking habits on respiratory health in the population. In 2005, legislation was implemented in Sweden to reduce smoking in restaurants, which probably contributed to the decrease in smoking. More women than men smoked already in 1996, and the decrease in smoking prevalence was more pronounced among men compared to women. Thus, the gender difference in smoking prevalence increased. Worryingly, a recent study indicates an increase in prevalence of smoking and symptoms of bronchitis among teen-age girls in Sweden [31]. It is of great importance to establish a decrease in cigarette consumption also among women; in particular since women are more susceptible to cigarette smoke than men [32].

Few studies have reported PAR of smoking for respiratory symptoms and asthma. Our results imply that if smoking had been eliminated, 10-25% of the symptom prevalence in the population would have been eliminated. A study comparing the Swedish part of the Global Allergy and Asthma European Network (GA2LEN) study performed in 2008 with the Swedish part of the European Community Respiratory Health Survey (ECHRS) from 1990 [5] also found a decrease in the prevalence of smoking, and a decrease in PAR of smoking for any wheeze. However, the methods for estimating PAR differed somewhat between the present study and the cited study.

Comparisons of prevalence estimates between studies are difficult, since populations, questionnaires, and classification of symptoms and diseases often differ. One major advantage of our study is the use of identical methods in both surveys. The same methods as in our study were used in Helsinki, Finland, and that study also found a decrease in symptoms of bronchitis parallel to a decrease in smoking prevalence from 1996 to 2006 [21]. In west Sweden, a decrease was seen in the prevalence of most respiratory symptoms from 1990 to 2008 parallel to a 50% decrease in smoking prevalence [6]. In contrast to our results and the studies cited above, a study in Stockholm using identical methods found the prevalence of symptoms to be more or less level from 1996 to 2006, despite a decrease in smoking prevalence by 43% [22].

The decrease in the prevalence of bronchitis symptoms is probably mainly a result of the decrease in smoking prevalence, but also of some other factor since we found a decrease among non-smokers. The decrease among non-smokers could be related to a reduction of environmental tobacco smoke (ETS), which followed the stricter Swedish smoking legislation. The increase in use of asthma medication, which can be prescribed also for bronchitis symptoms, may also contribute to a decrease in symptoms.

Wheeze is a common symptom both in asthma and bronchitis and is strongly related to smoking [6,22,25]. In contrast to bronchitis symptoms, the prevalence of any wheeze and asthmatic wheeze remained at a similar level in 2006 compared to 1996, also stratified by smoking habits, while recurrent wheeze decreased slightly but significantly. Since PAR of smoking was largest for recurrent wheeze, with smoking explaining about one fifth of the symptom prevalence, a decrease in the prevalence of recurrent wheeze was expected due to the decrease in smoking prevalence. Actually, a pronounced decrease in the prevalence of all wheeze symptoms was expected due to the decrease in smoking prevalence. One hypothesis that would explain the lack of an overall decrease in wheeze symptoms is that a parallel increase in asthma prevalence levels the prevalence of wheeze. Smoking has some anti-inflammatory effects and may be inversely related to allergic sensitisation [33,34]. Regarding asthma however, there is no data indicating a similar effect of smoking. Instead several studies suggest no effect or an increased risk of smoking on the incidence of asthma among adults [8-11]. Further, parental smoking increases the risk for childhood asthma [35,36]. Thus, it is not likely that the increase in asthma is related to the decrease in smoking.

Compared to similar studies from other countries [13,14,37] as well as in other Swedish studies using the same questionnaire as in our study [6,21,28], a higher prevalence of asthma was found in Norrbotten. This finding confirms previous indications of a north–south gradient in asthma prevalence in Sweden [38,39]. The prevalence of physician-diagnosed asthma increased from 1996 to 2006, also when stratified by smoking. The highest prevalence of asthma was found among ex-smokers, a result confirming other cross-sectional studies [6,12]. It is known that diagnosis of COPD significantly impacts smokers to quit smoking [40] and therefore it could be suggested that smokers who get an asthma diagnosis also are likely to quit smoking, as several studies have found an association between current smoking and incident asthma [8,10,11]. The prevalence of physician-diagnosed asthma decreased by age in both surveys, in line with results from other studies [6,22]. In 1996 however, in contrast to 2006, there was a higher prevalence of asthma in the oldest age group (60–69 years) compared to those aged 30–59 years. This result might reflect misclassification of COPD as asthma among the elderly in 1996.

Does the estimated increase in the prevalence of asthma reflect a “true” increase in asthma? The proportion reporting symptoms common in asthma decreased significantly from 1996 to 2006 among subjects with physician-diagnosed asthma. This decrease occurred despite the fact that the proportion of subjects using asthma medicines also decreased significantly among the asthmatics. These data suggest an increased awareness of asthma in the society, improved recognition of asthma in primary care and an increasing diagnostic activity, in particular of mild asthma, which probably have contributed to the observed increase in physician-diagnosed asthma; a result in line with studies in other areas [6,21,22]. On the other hand, the lack of decrease in asthmatic wheeze and any wheeze despite the major decrease in smoking prevalence may indicate a true increase in asthma in Northern Sweden. Also, both asthma and use of asthma medication increased most in the youngest age group, which imply a true increase of asthma since misclassification of asthma as e.g. COPD is unlikely among young subjects. One might argue that the observed increase in asthma could be due to both a true increase in asthma as well as an increased diagnostic activity, which would be a result in line with studies from other countries [41]. This hypothesis requires further studies including clinical examinations. In our study, the majority of subjects with physician-diagnosed asthma used asthma medicines in both surveys, although somewhat less so in 2006 compared to 1996. In a study from Australia the authors argue that improved guidelines on how to implement asthma treatment are required [42], but as we cannot provide detailed information about use of inhaled corticosteroids we cannot evaluate if the adherence to maintenance treatment is appropriate.

The validity and reliability of the present study was high due to the population-based samples, the high participation rates in both surveys and the use of a validated questionnaire. The somewhat lower participation rate in 2006 compared to 1996 could possibly lead to a slight overestimation of symptom prevalence in 2006 [43]. An overestimation in 2006 would not alter the findings of decreased prevalence in symptoms, but could possibly affect the observed increase in asthma prevalence. However, the close to identical prevalence estimates after adjustment to the age and gender distribution in Norrbotten each year verify the representativeness of the study sample. A weakness is the lack of clinical data when discussing asthma, and measures of cotinine for validating data about smoking. Further, data of exposure to ETS, body weight, gastro-oesophageal reflux, obstructive sleep apnoea and occupational dust exposure would have been valuable, since the presence of these factors may have changed between surveys, which in turn could have contributed to a change in both respiratory symptom and asthma prevalence. The cross-sectional study design weakens the discussion of causality. However, the strong and consistent association between smoking and respiratory symptoms in both surveys as well as the decrease of both smoking and bronchitis symptoms imply an improvement in respiratory health in the population at least partly due to the decrease in smoking.

## Conclusions

In conclusion, we found that respiratory symptoms, in particular bronchitis symptoms, decreased parallel to a decrease in smoking over a 10-year period from 1996 to 2006. In contrast, the prevalence of physician-diagnosed asthma increased during the same period. The hypothesis that the observed increase in asthma reflects a “true” increase in asthma prevalence has to be confirmed by clinical studies. Additionally, up to one fourth of the respiratory symptom prevalence in the population was attributable to smoking.

## Competing interests

None of the authors have any competing interests to declare.

## Authors’ contributions

HB carried out all data management and statistical analyses, interpreted data, drafted and finalized the manuscript. LH helped to draft the manuscript and interpret data. S-A J revised the manuscript. AL revised the manuscript and interpreted data. BL and ER designed and coordinated the study, revised the manuscript and contributed with important input to discussion. ER conceived the study and participated in the data collection process as well as supervised the analyses. All authors read and approved the final manuscript.
